# Relation between quantity and quality of peri-coronary epicardial adipose tissue and its underlying hemodynamically significant coronary stenosis

**DOI:** 10.1186/s12872-020-01499-w

**Published:** 2020-05-15

**Authors:** Yu Du, Lin Yang, Yan Liu, Bangguo Yang, Sai Lv, Chenping Hu, Yong Zhu, Hongkai Zhang, Qian Ma, Zhijian Wang, Yuyang Liu, Dongmei Shi, Yingxin Zhao, Lei Xu, Yujie Zhou

**Affiliations:** 1 Department of Cardiology, Beijing Anzhen Hospital, Capital Medical University, Beijing Institute of Heart Lung and Blood Vessel Disease, Beijing Key Laboratory of Precision Medicine of Coronary Atherosclerotic Disease, Clinical center for coronary heart disease, Capital Medical University, Beijing, 100029 China; 2 Department of Radiology, Beijing Anzhen Hospital, Capital Medical University, Beijing Institute of Heart Lung and Blood Vessel Disease, Beijing, 100029 China; 3 Department of Cardiology, Fuwai Yunnan Cardiovascular Hospital, Yunnan, 650000 China

**Keywords:** Epicardial adipose tissue, Coronary stenosis, Myocardial ischemia, Volume, Density

## Abstract

**Background:**

We aimed to investigate the association of lesion-specific epicardial adipose tissue (EAT) volume and density with the presence of myocardial ischemia.

**Methods:**

We enrolled 45 patients (55 lesions) with known or suspected coronary artery disease who underwent coronary computed tomography angiography (CTA) followed by invasive fractional flow reserve (FFR) assessment within 30 days. EAT volume (index) and density in patient-, vessel- and lesion-level were measured on CTA images. Lesion-specific ischemia was defined as a lesion with stenosis diameter > 90% or FFR ≤0.80. Multivariate analysis determined the independent association of EAT parameters with lesion-specific ischemia.

**Results:**

Mean age of the patients was 60 years, and 75% were male. Overall, 55.6% of patients had ischemic lesions and a mean FFR baseline value of 0.82 ± 0.10. Total EAT volume index was significantly higher in patients with functionally or anatomically significant stenosis. Specifically, peri-lesion EAT volume index, not the density, was positively correlated with lesion-specific ischemia independent of luminal stenosis and plaque characteristics (hazard ratio 1.56, 95% confidence interval 1.04–2.33, *P* = 0.032; per 0.1 ml/m^2^ increase). Moreover, peri-lesion EAT volume was negatively correlated with lesion FFR values, whereas total EAT volume was positively correlated with fat accumulation and glucose metabolism. In addition, there was no association of EAT volume or density with myocardial ischemia in vessel-level analysis.

**Conclusions:**

Lesion-specific EAT volume index, but not density, seems positively and independently associated with myocardial ischemia, while its incremental diagnostic value of lesion-specific ischemia should be further investigated.

## Background

Epicardial adipose tissue (EAT) is a specific fat depot between the myocardium and the visceral pericardium, mainly surrounding major epicardial coronary arteries or within the myocardium [1]. Numerous studies showed that the accumulation of EAT is closely associated with the presence and severity of coronary artery disease (CAD), myocardial ischemia, plaque vulnerability, and major adverse cardiovascular events (MACE) [2–5]. However, not all patients with increased EAT volume develop CAD and vice versa.

To further explain the relation between EAT and CAD, the following two factors are attracting much attention. The first one is peri-coronary EAT, because there is close cross-talk between this focal metabolo-active EAT and its underlying coronary wall via a paracrine pathway [6]. External administration of interleukin 1β or monocyte chemotactic protein 1 to porcine coronary arteries led to coronary wall inflammation and atherosclerosis formation [7, 8], while surgical removal of peri-coronary EAT ameliorated the progression of coronary atherosclerosis in the pigs [9]. The second important factor is EAT quality, shown as fat attenuation on computed tomography (CT), which reveals adipocyte lipid content and size, reflecting a metabolic response to cardiovascular risk factors [10]. Antonopoulos et al. found that the peri-coronary EAT attenuation index can expose vascular inflammation, subclinical coronary atherosclerosis, and vulnerable plaque [11] and even independently predict cardiac mortality [12].

Accordingly, it is theoretically feasible to determine the hemodynamic significance and vulnerability of coronary stenosis by simply measuring peri-coronary EAT quantity or quality via coronary computed tomography angiography (CTA) in high-risk CAD patients before hospitalization. Therefore, in this hypothesis-generating study, we aimed to investigate the association of lesion-specific EAT volume or attenuation with the functional significance of coronary stenosis on a hospital-based Chinese population.

## Methods

### Study patients

We retrospectively screened 134 consecutive patients who underwent coronary CTA and invasive fractional flow reserve (FFR) assessment for known or suspected CAD between June 2015 and June 2018 in our center. Exclusion criteria included the presence of acute coronary syndrome (ACS) within the past 30 days; a history of myocardial revascularization or old myocardial infarction; presence of co-morbidities (i.e., heart failure, atrial fibrillation, insulin-dependent diabetes, severe lipoprotein disorders, chronic liver or renal disease, inflammatory disease, malignancy); coronary CTA not performed within 30 days before FFR evaluation or technical failure to measure FFR or EAT parameters; presence of three-vessel disease, left main or chronic totally occluded lesions, or any intermediate lesions on main branch not measured by FFR. In addition, in lesion-level analysis, the following FFR-measured lesions were excluded: ostial lesions; lesions in a distal segment of a vessel; bifurcation lesions; side-branch lesions; extensively calcified or tortuous lesions. The study protocol conformed to the Declaration of Helsinki and was approved by the ethics committee of Beijing Anzhen Hospital of Capital Medical University. Written informed consent was obtained from each patient.

### CT imaging

CT imaging was performed using a dual-source 128-slice CT scanner (Somatom Definition Flash, 280-ms rotation, 2 × 128 × 0.6 collimation; Siemens Healthcare, Forchheim, Germany). Contrast-enhanced scanning and reconstruction protocols were as previously described [13]. In this study, we enrolled only patients with contrast-enhanced scans obtained at 120-kV tube voltage and with a z-axis scan range at least from the pulmonary artery bifurcation to the ventricular apex. We obtained the location, length, volume, and classification of coronary plaque on CTA images. Plaque was classified as calcified or non-calcified. Calcified plaque referred to lesions composed exclusively of structures with a CT attenuation higher than that of the contrast-enhanced coronary lumen in at least two planes. Non-calcified plaque was defined as lesions clearly assignable to the vessel wall (in at least two views) with an attenuation lower than that of the contrast-enhanced lumen.

### EAT volume and density quantification

**Fig. 1:**
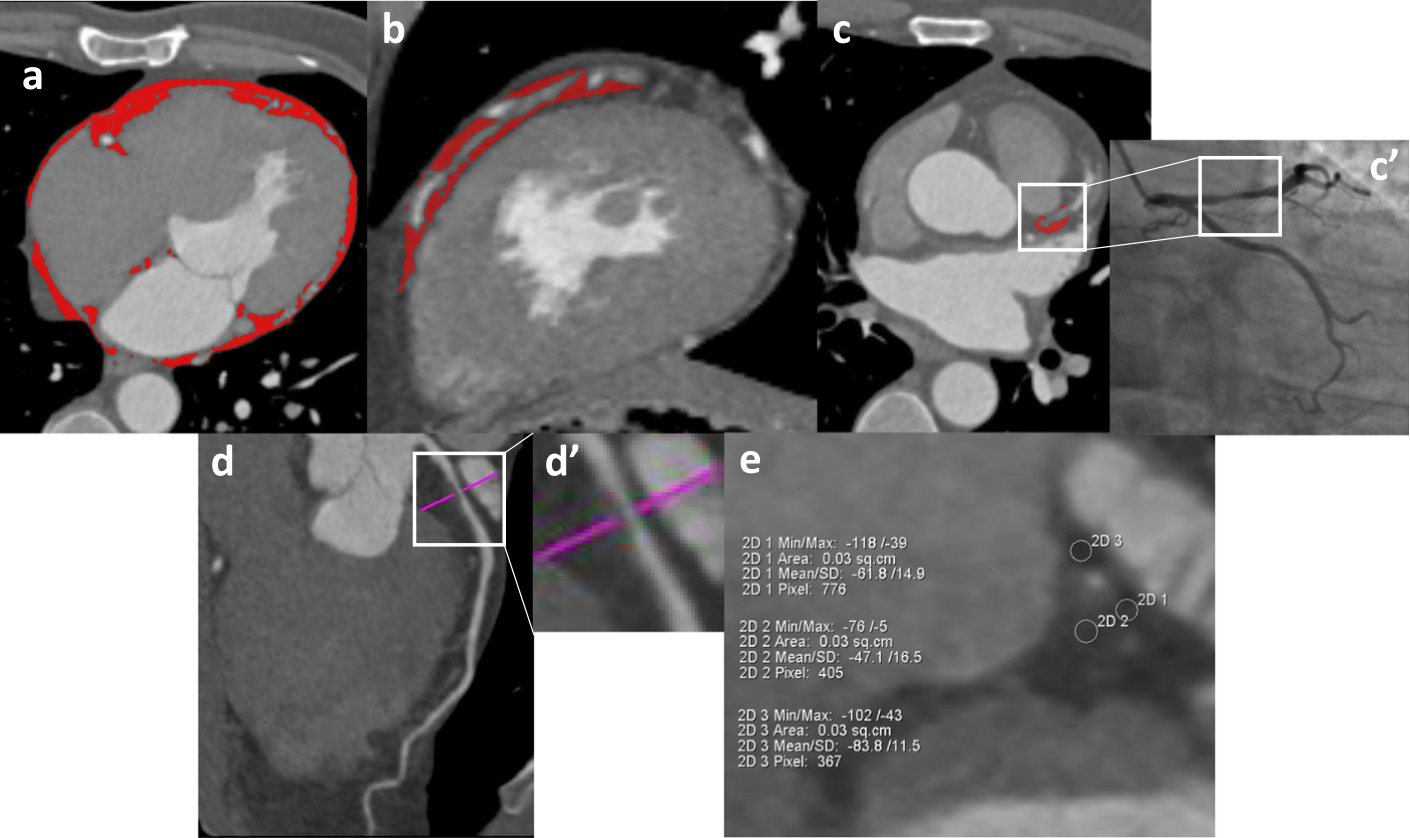
Measurements of EAT quantity and quality on coronary CTA images. Cross-sectional image for volume measurement of total EAT (**a**), peri-vessel EAT (**b**), and peri-lesion EAT (**c**). Location and length of study coronary lesion were double checked using CTA (**c**) and corresponding angiographic images (**c′**). ROIs are stained red. The narrowest location in diameter stenosis of study lesion were determined in (**d**) and (**d'**). According to the most severe diameter stenotic cross-sectional CTA image (**e**), peri-lesion EAT density was the average CT attenuation of peri-lesion EAT. EAT, epicardial adipose tissue; CTA, computed tomography angiography; ROI, region of interest

EAT volume and density were quantified offline from CTA images using cardiac risk analysis software (Siemens, Erlangen, Germany) and a computer workstation (MMWP, Siemens Healthcare). CT attenuation range of EAT was set between − 190 Hounsfield Unit (HU) and − 30 HU. Assessment of total EAT (ranging from the bifurcation of the pulmonary artery to the end slice of the left ventricular apex, from the outer wall of the myocardium to the visceral pericardium) volumes were according to region of interests (ROIs) on all axial imaging planes that were semi-automatically delineated (every 5 mm interval), and then manually adjusted to modify and track the silhouette of the EAT on every slice (Fig. 1a) [14]. Peri-vessel EAT (veEAT) (surrounding from the ostium to the end of each major epicardial coronary artery within the interventricular or atrioventricular groove, respectively) volumes were manually delineated according to ROIs on all axial imaging slices (every 5 mm interval) (Fig. 1b) [15]. While, peri-lesion EAT (leEAT) was a part of veEAT, which covered the vessel segment containing the full length of study coronary lesion, and leEAT volumes were calculated by ROIs on each axial imaging slice (every 1 mm interval) (Fig. 1c) [16]. Volume index was defined as volume (ml) divided by body surface area (BSA, m^2^).

We also obtained leEAT density by using manual measurement, defined as the mean value of three or more circular ROIs (each ROI area = 0.03 cm^2^) surrounding the cross-sectional slice of the study lesion with the most severe diameter stenosis (Fig. 1d, d'). The location of ROIs was required to be more than 1 mm from the adventitia of the coronary artery to reduce the influence of luminal contrast on leEAT density (Fig. 1e) [14]. Correspondingly, the veEAT density—defined as EAT CT attenuation of the proximal and distal reference segments adjacent to the stenotic segment and free from stenosis—was measured and used as the reference veEAT density. All EAT parameter measurements were performed repeatedly by an experienced CT reader, who was blinded to the clinical characteristics of the patients and the functional significance of the coronary stenosis. Intra-observer reproducibility was excellent [intraclass correlation coefficient 0.95, 95% confidence interval (CI) 0.90–0.97, *P* <  0.001].

### Coronary angiography and fractional flow reserve

All patients underwent coronary angiography according to standard clinical protocols. CAD was diagnosed by the presence of at least one coronary artery stenosed > 50% in diameter stenosis on coronary angiography. Stenosis causing 30–90% coronary lumen reduction were evaluated by invasive FFR. A lesion with luminal stenosis ≥75% was defined as significantly stenosis. A lesion diameter with > 90% stenosis or FFR ≤0.80 indicated hemodynamic significance. Invasive FFR was measured using a coronary pressure wire (Volcano, Rancho Cordova, CA, USA or St Jude Medical, Minneapolis, MN, USA), as described previously [17]. In this study, maximum hyperemia was induced by intravenous administration of adenosine triphosphate (140–180 μg/kg/min) via the forearm vein. The decision for revascularization was left to the operator’s discretion.

### Statistical analysis

Continuous variables were expressed as means ± standard deviation, or the median (lower quartile, upper quartile), where indicated. Mean values were compared using Student’s t test and median values using the Mann–Whitney U test. Categorical variables were expressed as percentages and analyzed using the chi-square test or Fisher's exact test where appropriate. The correlations of EAT parameters with clinical characteristics were evaluated using Spearman’s correlation analysis. Univariate and multivariate analyses were performed to determine the independent predictors for hemodynamically significant coronary stenosis. *P* <  0.05 was considered to indicate statistical significance. All statistical analyses were performed using SPSS 22.0 software (IBM, Armonk, NY, USA).

## Results

A total of 45 patients were enrolled in patient-level analysis. The study flow chart is shown in Supplementary Figure S1. As shown in Table 1, the mean age of the patients was 60 years, 75% were male, and 67% had one-vessel disease. The mean time between coronary CTA and invasive angiography and FFR was 11 days. In all, 68 lesions had a diameter stenosed ≥30%. Among them, FFR was performed on 65 (96%) lesions. Three lesions with diameters stenosis > 90% were not measured by FFR. After screening, 55 lesions were enrolled in lesion-level analysis. As shown in Table 2, most of lesions were located in the left anterior descending (LAD) coronary artery (71%) or its middle segments (66%). Nearly half of the lesions had intermediate stenosis (49%), and 62% had calcified plaque. The mean preoperative FFR value was 0.82. Ischemic lesions accounted for 47%, and eventually 29% of patients (22% of lesions) underwent percutaneous coronary intervention (PCI).

**Table 1:** Baseline characteristics of study patients according to the presence of ischemia lesion

	Total (*n* = 45)	Non-ischemia patient(*n* = 20)	Ischemia patient (*n* = 25)	*P* value
**Clinical variable**				
Age (years)	59.6 ± 4.7	58.2 ± 4.8	60.7 ± 4.4	0.073
Male	34 (75.6)	13 (65.0)	21 (84.0)	0.176
Body weight (kg)	73.8 ± 11.1	74.7 ± 13.5	73.1 ± 9.0	0.649
Height (m)	1.69 ± 0.08	1.69 ± 0.09	1.70 ± 0.07	0.689
BMI (kg/m^2^)	25.7 ± 2.8	26.1 ± 3.2	25.4 ± 2.4	0.382
BSA (m^2^)	1.86 ± 0.18	1.86 ± 0.21	1.85 ± 0.14	0.818
Hypertension	26 (57.8)	10 (50.0)	16 (64.0)	0.379
Diabetes mellitus	13 (28.9)	3 (15.0)	10 (40.0)	0.100
Hypercholesteromia	15 (33.3)	6 (30.0)	9 (36.0)	0.757
Current smoking	17 (37.8)	5 (25.0)	12 (48.0)	0.135
PAD or stroke	3 (6.7)	2 (10.0)	1 (4.0)	0.577
Family history of CAD	5 (11.1)	2 (10.0)	3 (12.0)	1.000
LVEF (%)	65.2 ± 5.3	66.4 ± 5.6	64.2 ± 5.0	0.181
**Procedural variable**				
Time interval (day)	11.3 ± 8.4	12.9 ± 9.2	10.1 ± 7.7	0.304
No. of diseased vessels				0.005
0-vessel disease ^a^	2 (4.4)	2 (10.0)	0	
1-vessel disease	30 (66.7)	17 (85.0)	13 (52.0)	
2-vessel disease	7 (15.6)	1 (5.0)	6 (24.0)	
3-vessel disease	6 (13.3)	0	6 (24.0)	
No. of lesions per-patient	1.51 ± 0.73	1.15 ± 0.37	1.80 ± 0.82	0.003
No. of ischemia lesions per-patient	0.67 ± 0.71	0	1.20 ± 0.50	< 0.001
No. of significantly stenotic lesions per-patient	0.47 ± 0.59	0.15 ± 0.37	0.72 ± 0.61	0.001
No. of significantly stenotic patients	19 (42.2)	3 (15.0)	16 (64.0)	0.002
Coronary intervention	13 (28.9)	0	13 (52.0)	< 0.001
No. of stents	0.36 ± 0.61	0	0.64 ± 0.70	< 0.001
Length of stents	9.04 ± 15.66	0	16.3 ± 18.1	< 0.001
**EATparameter**				
Total EAT volume (ml)	89.6 (73.4, 122.8)	84.0 (64.7, 110.5)	102.0 (74.7, 139.8)	0.077
Total EAT volume index (ml/m^2^)	49.8 (40.5, 64.4)	47.6 (35.1, 55.4)	53.4 (43.1, 71.6)	0.032
**Medications on discharge**				
Aspirin	42 (93.3)	18 (90.0)	24 (96.0)	0.577
Clopidogrel	27 (60.0)	11 (55.0)	16 (64.0)	0.760
Statin	37 (82.2)	15 (75.0)	22 (88.0)	0.435
ACEI/ARB	18 (40.0)	6 (30.0)	12 (48.0)	0.359
Nitrates	28 (62.2)	8 (40.0)	20 (80.0)	0.012

*BMI* body mass index, *BSA* body surface area, *PAD* peripheral artery disease, *CAD* coronary artery disease, *LVEF* left ventricular ejection fraction, *EAT* epicardial adipose tissue, *ACEI* angiotensin-converting enzyme inhibitor, *ARB* Angiotensin II receptor blocker

^a^indicated at least one lesion with angiographic diameter stenosis of 30–49% in major epicardial coronary artery

**Table 2:** Baseline characteristics of study lesions according to the presence of ischemia

	Total (*n* = 55)	Non-ischemia lesion (*n* = 29)	Ischemia lesion (*n* = 26)	*P* value
**Lesion parameter**				
Location				0.350
LAD	39 (70.9)	18 (62.1)	21 (80.8)	
LCX	4 (7.3)	3 (10.3)	1 (3.8)	
RCA	12 (21.8)	8 (27.6)	4 (15.4)	
Segment				0.584
Proximal	19 (34.5)	9 (31.0)	10 (38.5)	
Middle	36 (65.5)	20 (69.2)	16 (61.5)	
Reference vessel diameter (mm)				
Proximal	3.9 ± 0.5	4.0 ± 0.5	3.9 ± 0.5	0.341
Distal	3.0 ± 0.5	3.1 ± 0.6	2.8 ± 0.4	0.069
Stenosis severity				< 0.001
30 ~ 49%	6 (10.9)	6 (20.7)	0	
50 ~ 74%	27 (49.1)	18 (62.1)	9 (34.6)	
≥ 75%	22 (40.0)	5 (17.2)	17 (65.4)	
Plaque length (mm)	14.2 (11.1, 20.2)	12.9 (10.8, 16.7)	19.3 (12.0, 21.5)	0.028
Plaque volume (mm^3^)	139.0 (81.0, 192.0)	118.0 (76.0, 161.0)	156.5 (84.8, 220.3)	0.159
Plaque type				0.405
Non-calcified	21 (38.2)	13 (44.8)	8 (30.8)	
Calcified	34 (61.8)	16 (55.2)	18 (69.2)	
**EAT parameter**				
veEAT volume (ml)	7.14 (4.93, 9.18)	7.20 (4.98, 8.54)	6.89 (4.75, 10.57)	0.607
veEAT volume index (ml/m^2^)	3.80 (2.62, 4.70)	3.80 (2.67, 4.26)	3.83 (2.38, 5.87)	0.508
leEAT volume (ml)	0.57 (0.35, 0.72)	0.50 (0.32, 0.66)	0.64 (0.37, 1.07)	0.051
leEAT volume index (ml/m^2^)	0.30 (0.20, 0.40)	0.27 (0.19, 0.35)	0.34 (0.20, 0.53)	0.045
veEAT density (HU)				
Proximal reference vessel	−72.91 ± 10.56	−71.94 ± 9.60	−74.00 ± 11.63	0.475
Distal reference vessel	−90.86 ± 11.82	−90.40 ± 9.65	−91.38 ± 14.04	0.763
leEAT density (HU)	−77.32 ± 11.63	−76.54 ± 12.71	−78.23 ± 10.44	0.600
**Procedural variable**				
Pre-procedural FFR ^a^	0.82 ± 0.10	0.89 ± 0.05	0.74 ± 0.08	< 0.001
Coronary intervention	12 (21.8)	0	12 (46.2)	< 0.001
No. of stents per-lesion	0.24 ± 0.47	0	0.50 ± 0.58	< 0.001
Length of stents	6.38 ± 12.91	0	13.50 ± 16.12	< 0.001
Diameter of stents	0.72 ± 1.38	0	1.52 ± 1.69	< 0.001

*LAD* left anterior descending, *LCX* left circumflex, *RCA* right coronary artery, *EAT* epicardial adipose tissue, *veEAT* peri-vessel epicardial adipose tissue, *leEAT* peri-lesion epicardial adipose tissue, *FFR* fractional flow reserve

^a^indicated FFR was not measured in three lesions of the ischemia group because of angiographically severe stenosis

### EAT volume and myocardial ischemia in patient-level analysis

**Fig. 2:**
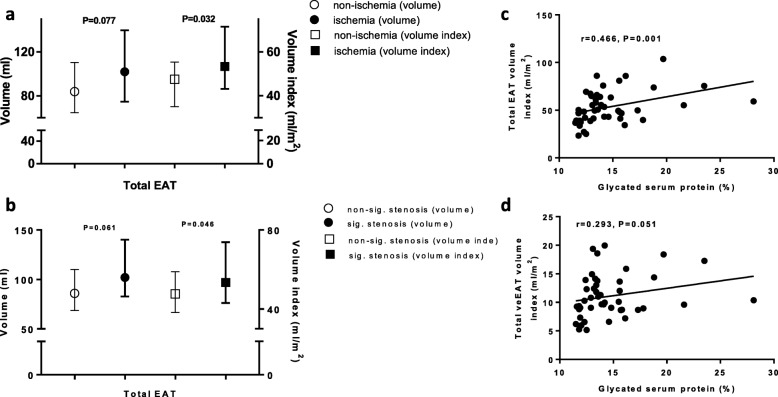
EAT volume and myocardial ischemia in patient-level analysis. Total EAT volume (index) in patient with functionally (**a**) or anatomically significant stenosis (**b**). Correlations of glycated serum protein with total EAT volume index (**c**) and total veEAT volume index (**d**). EAT, epicardial adipose tissue; veEAT, peri-vessel epicardial adipose tissue

Patients were divided into non-ischemia and ischemia groups based on the presence of at least one hemodynamically relevant lesion (44 and 56%, respectively). The ischemic patients were slightly older than the non-ischemic patients, had more extensive and severe CAD, and half underwent PCI (Table 1). As shown in Fig. 2a, the total EAT volume was non-significantly greater in ischemic patients than non-ischemic patients (102.0 vs 84.0 ml, *P* = 0.077), whereas this pattern became statistically significant regarding the indexed total EAT volume (53.4 vs 47.6 ml/m^2^, *P* = 0.032). Patients were also divided into non-significant and significant stenosis groups according to the presence of at least one significantly stenotic lesion (58 and 42%, respectively). Similarly, as shown in Fig. 2b, compared with non-significant stenosis patients, significant stenosis patients had a slightly higher total EAT volume (102.0 vs 85.9 ml, *P* = 0.061) and a markedly higher indexed total EAT volume (53.4 vs 47.6 ml/m^2^, *P* = 0.046). The relation of EAT volume and myocardial ischemia in a dose-dependent manner was not further investigated because most of the patients had at least one ischemic or significant stenosis (91 and 95%, respectively).

In addition, we found that the total EAT volume was positively correlated with the body mass index (r = 0.381, *P* = 0.010), fasting blood glucose level (r = 0.341, *P* = 0.022), and glycated serum protein level (r = 0.434, *P* = 0.003). Moreover, the indexed total EAT volume was still positively correlated with the fasting blood glucose (r = 0.330, *P* = 0.027) and glycated serum protein (r = 0.466, *P* = 0.001) (Fig. 2c). Glycated serum protein was also positively correlated with the total veEAT volume (r = 0.308, *P* = 0.040) and its index value (r = 0.293, *P* = 0.051) (Fig. 2d).

### EAT volume or density and myocardial ischemia in lesion-level analysis

**Fig. 3:**
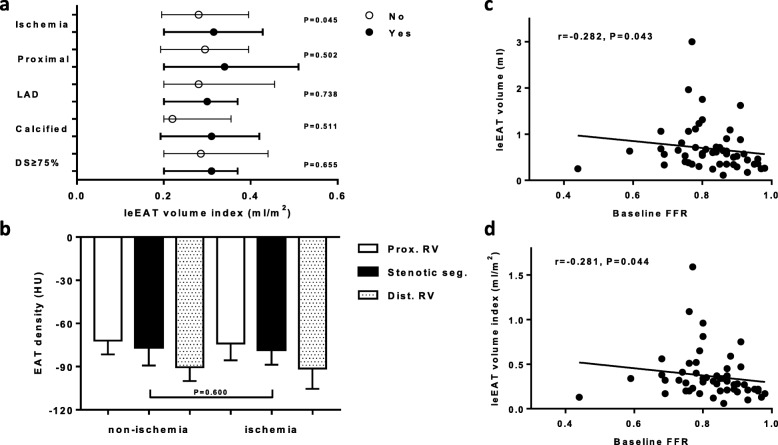
The leEAT volume or density and lesion-specific characteristics. (**a**) The leEAT volume index and lesion-specific characteristics. (**b**) EAT density and lesion ischemia. Correlations of baseline FFR values with leEAT volume (**c**) and its indexed value (**d**). leEAT, peri-lesion epicardial adipose tissue; LAD, left anterior descending coronary artery; DS, diameter stenosis; EAT, epicardial adipose tissue; FFR, fractional flow reserve

All study lesions were divided into non-ischemic and ischemic lesions, the baseline characteristics of which are shown in Table 2. Compared with non-ischemia lesions, ischemic lesions had more severe diameter stenosis and greater plaque length. Pre-procedural FFR values for ischemic lesions were significantly lower than those of the non-ischemic lesions (0.74 ± 0.08 vs 0.89 ± 0.05, *P* < 0.001). Ischemic lesions accounted for 46% of all lesions that underwent PCI. None of the non-ischemic lesions were treated. As shown in Table 2, compared with non-ischemic lesions, ischemic lesions had higher leEAT volumes (0.64 vs 0.50 ml, P = 0.051) and a significantly higher leEAT volume index (0.34 vs 0.27 ml/m^2^, *P* = 0.045). This pattern, however, was not apparent for the veEAT volume (6.89 vs 7.20 ml, *P* = 0.607) or veEAT volume index (3.83 vs 3.80 ml/m^2^, *P* = 0.508). Subgroup analysis showed that, except for the ischemic lesions (P = 0.045), the leEAT volume index was not significantly different with respect to proximal lesions, LAD, calcified plaque, or diameter stenosed ≥75% (all *P* > 0.05) (Fig. 3a). Furthermore, as shown in Supplementary Table S1, the independent lesion-level predictors for ischemia were diameter stenosis of ≥75% [hazard ratio (HR) 14.56, 95% CI 2.93–72.24, *P* = 0.001] and the leEAT volume index (per 0.1 ml/m^2^ increase) (HR 1.56, 95% CI 1.04–2.33, *P* = 0.032), after adjusting for LAD, proximal segment, plaque length, plaque calcification, and reference vessel diameter.

We also evaluated EAT density between non-ischemic and ischemic lesions, which showed no significant differences with regard to leEAT density (− 76.54 ± 12.71 vs − 78.23 ± 10.44 HU, *P* = 0.600), veEAT density of the proximal reference vessel (− 71.94 ± 9.60 vs − 74.00 ± 11.63 HU, *P* = 0.475)], or the distal reference vessel (− 90.40 ± 9.65 vs − 91.38 ± 14.04 HU, *P* = 0.763) (Fig. 3b). Additionally, we found that baseline FFR values negatively correlated with the leEAT volume (r = − 0.282, *P* = 0.043) (Fig. 3c) or the leEAT volume index (r = − 0.281, *P* = 0.044) (Fig. 3d).

## Discussion

We performed a proof-of-concept study to evaluate the associations of EAT volume and density with myocardial ischemia in patient-, vessel-, and lesion-level analyses, in a high-risk Chinese population. First, we found that the total EAT volume index was markedly increased in patients with functionally or anatomically significant stenosis. Second, leEAT volume index was markedly higher for ischemic lesions, furthermore, this index was a lesion-specific predictor of hemodynamic relevance, independent of a variety of lesion-level parameters. Third, the total EAT volume was positively correlated with fat accumulation and glucose metabolism, whereas the leEAT volume was negatively correlated with lesion FFR values. Fourth, we did not observe any significant differences in CT attenuation between non-ischemic and ischemic lesions in both lesion- or vessel-level analyses.

The significant correlations of the total EAT volume with functionally or anatomically significant CAD have been widely observed [2, 3], although not reported in a recent study by Muthalaly et al. with moderately severe CAD patients [18] or even in the prospective, large-scale CORE320 multicenter study [19]. Likewise, Romijn et al. found an independent relation between EAT volume and myocardial ischemia, but the diagnostic performance for identifying hemodynamically significant CAD was not improved when considering coronary artery calcification [20]. A recent meta-analysis that included more than 40,000 subjects at low to intermediate risk of cardiovascular disorders showed that EAT volume was an independent predictor of obstructive stenosis, significant stenosis, myocardial ischemia, and MACE, irrespective of traditional cardiovascular risk factors [21]. In line with this meta-analysis, we found that indexed total EAT volume was significantly increased in patients with functionally or anatomically significant stenosis. The above conflicting results might be attributed, at least in part, to a diversity of populations [21], more importantly illustrate a complex relation between EAT and the pathogenesis of CAD, which is hardly explain by the overall EAT volume alone.

Recent evidence suggests a potentially active role of peri-coronary EAT in the pathogenesis of coronary atherosclerosis [22]. It was reported that peri-coronary segment EAT quantity was related to plaque size and composition, independent of cardiovascular risk factors and overall EAT volume [16, 23]. Also, this coronary segment-specific EAT volume was associated with luminal stenosis severity, the presence of reversible perfusion defects, and the culprit lesion [15, 24, 25]. Unlike the above studies, we performed a lesion-specific analysis and found a close relation between indexed leEAT volume and the presence of lesion ischemia. Furthermore, every 0.1 ml/m^2^ increase in the leEAT volume index was independently associated with a 1.6-fold increased risk of lesion ischemia, irrespective of lesion stenosis or plaque features. Although the predicting value was relatively small, our results showed the feasibility to predict lesion-specific ischemia using leEAT volume index, which appeared to have a promising prospective considering a large and increasing number of patients referred to undergo coronary CTA examination for suspected CAD. We did not, however, observe a markedly increased leEAT volume index in significantly luminal stenosis or non-calcified plaque, which has been reported elsewhere [24]. Mahabadi et al., however, also found no difference between non-calcified and calcified plaque in terms of leEAT volume [16].

Additionally, a vessel-level analysis showed no associations of veEAT volume and ischemia, which might support the hypothesis that the function of EAT varies depending on its particular depot. In line with this notion, we found the total EAT volume in patient- or vessel-level was significantly associated with fat accumulation and glucose metabolism, while the leEAT volume was associated with FFR values. More specifically, a dysfunctional secretion profile and M2 macrophage accumulation in lesion-level analysis were reported to be higher for peri-coronary EAT near stenotic coronary segments than for those near non-stenotic segments [26, 27]. Another possible explanation is that the metabolic activities of veEAT and leEAT, shown as EAT CT attenuation, were not consistent or remained unchanged. Markedly increased peri-coronary EAT CT attenuation was observed in stenotic segments, lesions with ^18^F-sodium fluoride uptake on positron emission tomography and culprit lesions [28–30]. In this study, however, there were no significant differences between ischemic and non-ischemic lesions in terms of peri-coronary EAT and peri-vessel EAT attenuation. In line with our findings, Hell et al. found that EAT volume, but not density, was associated with myocardial ischemia in patients suspected of having CAD [31]. Also, Balcer et al. reported that the peri-coronary EAT volume, but not attenuation, was independently correlated with culprit lesions [25].

This study had some limitations. First, the study’s sample size was relatively small, which impeded the performance of more subgroup or exploratory analyses. Second, because of the relatively strict inclusion and exclusion criteria, extrapolating our conclusions to other populations should be cautious. Third, based on the cross-sectional design, no causal relation could be confirmed, although multivariate regression analysis was performed. Fourth, methods for measuring the peri-coronary fat quality varies among studies, which might lead to non-comparable results among them. In addition, ROIs of veEAT and leEAT were manually, not automatically, delineated due to software limitations.

## Conclusion

We found that the indexed lesion-specific EAT volume appeared to be positively associated with lesion ischemia, independent of luminal stenosis and plaque characteristics. Hence, the peri-coronary lesion EAT volume index might be a promising marker for identifying lesion-specific ischemia, although its incremental diagnostic value when considering other risk factors should be further investigated.

## Supplementary information

**Additional file 1: Figure S1.** Study flow-chart. **Table S1.** Lesion-level predictors for myocardial ischemia in univariate and multivariate analyses.

## Data Availability

The data that support the findings of this study are available on request from the corresponding author.

## Abbreviations

EAT: Epicardial adipose tissue
CAD: Coronary artery disease
MACE: Major adverse cardiovascular events
CT: Computed tomography
CTA: Computed tomography angiography
FFR: Fractional flow reserve
ACS: Acute coronary syndrome
ROI: Regions of interest
HU: Hounsfield unit
veEAT: Peri-vessel EAT
leEAT: Peri-lesion EAT
BSA: Body surface area
CI: Confidence interval
LAD: Left anterior descending
PCI: Percutaneous coronary intervention
HR: Hazard ratio
