# Supplementary material for: Relation between quantity and quality of peri-coronary epicardial adipose tissue and its underlying hemodynamically significant coronary stenosis
Source: BMC Cardiovasc Disord. 2020 May 15;20:226. doi: 10.1186/s12872-020-01499-w (PMC7227353; doi:10.1186/s12872-020-01499-w)
Supplement: Supplementary file 1 — Additional file 1: Figure S1. Study flow-chart. Table S1. Lesion-level predictors for myocardial ischemia in univariate and multivariate analyses. [file 12872_2020_1499_MOESM1_ESM.docx]

**SUPPLEMENTARY DATA**

Relation between quantity and quality of peri-coronary epicardial adipose tissue and its underlying

hemodynamically significant coronary stenosis

Yu Du, MD, Lin Yang, MD, Yan Liu, MD, Bangguo Yang, MD, Sai Lv, MD, Chenping Hu, MD,Yong Zhu, MD,Hongkai Zhang, MD, Qian Ma, MD, PhD, Zhijian Wang, MD, Yuyang Liu, MD, Dongmei Shi, MD, Yingxin Zhao, MD, Lei Xu, MD, Yujie Zhou, MD, PhD


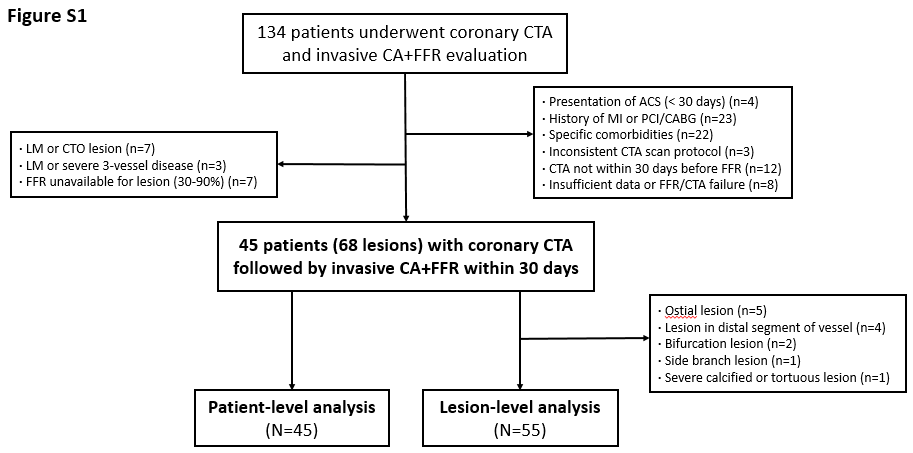


**Figure S1.** Study flow-chart. CTA, computed tomography angiogram; CA, coronary angiograph; FFR, fractional flow reserve; ACS, acute coronary syndrome; MI, myocardial infarction; PCI, percutaneous coronary intervention; CABG, coronary artery bypass grafting; LM, left main; CTO, chronic total occlusion.

**Table S1. Lesion level predictors for ischemia in univariate and multivariate analyses.**

| **Variable** | **Univariate Analysis** | | |  | **Multivariate Analysis** | | |
| --- | --- | --- | --- | --- | --- | --- | --- |
|  | **HR** | **95% CI** | **P** |  | **HR** | **95% CI** | **P** |
| LAD | 2.57 | 0.75-8.79 | 0.133 |  | 1.67 | 0.26-10.95 | 0.592 |
| Proximal segment | 1.39 | 0.46-4.24 | 0.564 |  | 1.06 | 0.21-5.37 | 0.942 |
| DS≥75% | 9.07 | 2.58-31.88 | 0.001 |  | 14.56 | 2.93-72.24 | 0.001 |
| Plaque length | 1.11 | 1.02-1.22 | 0.023 |  | 1.10 | 0.99-1.23 | 0.083 |
| Plaque volume | 1.004 | 0.997-1.010 | 0.298 |  |  |  |  |
| Plaque calcification | 1.83 | 0.60-5.54 | 0.286 |  | 1.34 | 0.26-6.91 | 0.729 |
| Proximal RVD | 0.59 | 0.20-1.74 | 0.336 |  |  |  |  |
| Distal RVD | 0.37 | 0.12-1.14 | 0.083 |  | 0.42 | 0.10-1.78 | 0.236 |
| leEAT volume index  (per 0.1 ml/m^2^ increase) | 1.37 | 1.01-1.87 | 0.042 |  | 1.56 | 1.04-2.33 | 0.032 |

HR, hazard ratio; CI, confidence interval; LAD, left anterior descending; DS, diameter stenosis; RVD, reference vessel diameter; leEAT, peri-lesion epicardial adipose tissue.
